# The Language of Glove: Wireless gesture decoder with low-power and stretchable hybrid electronics

**DOI:** 10.1371/journal.pone.0179766

**Published:** 2017-07-12

**Authors:** Timothy F. O’Connor, Matthew E. Fach, Rachel Miller, Samuel E. Root, Patrick P. Mercier, Darren J. Lipomi

**Affiliations:** 1 Department of NanoEngineering, University of California, San Diego, 9500 Gilman Drive, La Jolla, CA, United States of America; 2 Department of Electrical and Computer Engineering, University of California, San Diego, 9500 Gilman Drive, La Jolla, CA, United States of America; Universita del Salento, ITALY

## Abstract

This communication describes a glove capable of wirelessly translating the American Sign Language (ASL) alphabet into text displayable on a computer or smartphone. The key components of the device are strain sensors comprising a piezoresistive composite of carbon particles embedded in a fluoroelastomer. These sensors are integrated with a wearable electronic module consisting of digitizers, a microcontroller, and a Bluetooth radio. Finite-element analysis predicts a peak strain on the sensors of 5% when the knuckles are fully bent. Fatigue studies suggest that the sensors successfully detect the articulation of the knuckles even when bent to their maximal degree 1,000 times. In concert with an accelerometer and pressure sensors, the glove is able to translate all 26 letters of the ASL alphabet. Lastly, data taken from the glove are used to control a virtual hand; this application suggests new ways in which stretchable and wearable electronics can enable humans to interface with virtual environments. Critically, this system was constructed of components costing less than $100 and did not require chemical synthesis or access to a cleanroom. It can thus be used as a test bed for materials scientists to evaluate the performance of new materials and flexible and stretchable hybrid electronics.

## Introduction

This paper describes a sensor glove capable of converting hand gestures to text wirelessly using American Sign Language (ASL), and of controlling a virtual hand. The components of the entire system cost less than $100.00 (S1 Fig), excluding the cost of the laptop or smartphone. Low-cost, experimentally accessible platforms can allow laboratories to accelerate the discovery of materials designed to be integrated into whole devices in realistic scenarios.[1][2] The key enabling feature of the system is the use of a solution-processed, commercial conductive fluoroelastomer as a strain gauge that is stable over several months in the ambient air and over a thousand strain cycles. It is our hope that this material could play a role in stretchable hybrid electronics similar to the role played by poly(dimethylsiloxane) (PDMS) in soft lithography[3] and soft robotics.[4] That is, an inexpensive, commercially available material with reproducible properties in prototype devices.

Currently, the primary methods for tracking the positions of the human body[5] are through optical systems, by using an electromagnetic field, or by employing arrays of wearable sensors.[6] Optical systems comprising infrared emitters and receivers, in particular, have been successfully developed into systems for virtual reality and biomechanical analysis.[7][8] While these systems have low latencies and high spatial resolution, they require expensive and immovable infrastructure. Cameras, which make systems for tracking mobile in either the visible or infrared regimes, have also been successfully implemented,[9] but such systems need to be positioned away from the human body, sometimes awkwardly to maintain line-of-sight, and further have large power requirements for sophisticated image acquisition and processing hardware. A portable, wearable system, in contrast, does not have these constraints. We chose a glove as the test-bed system because it is the archetypal form factor for intuitive human-machine interfaces. That is, unlike other remote controls (e.g., a mouse, game controller, keyboard, and joystick), gloves interface directly with human hands. A gesture-tracking glove could thus enable a more seamless interface for consumer electronics, virtual and augmented reality, telesurgery,[10] technical training,[11] and wearable devices for covert operations—from piloting areal drones[12] to controlling bomb-diffusing robots.[13]

Many approaches have been used to develop wearable strain sensors and to integrate them with computation and communication. Stretchable piezoresistive strain sensors made from patterned silicon nanomembranes,[14] composite nanomaterials,[15] conjugated polymers,[16] graphene,[17] and many other material systems[18] possess a number of desirable qualities such as ultra-thinness, flexibility or stretchablility,[19] or ease of fabrication by printing.[20] Work has begun to develop more complex systems that integrate stretchable circuitry,[21][22] sensing,[23] computation,[24] and communication,[25] as human-machine interfaces using systems of advanced materials. These systems employ pressure and capacitive transducers made of microcracked gold to measure the articulation of fingers,[26] composites of gold nanowires and polyaniline to control a robot arm,[15] patterned graphene heterostructures[27] and silver nanowires to control a virtual hand,[28] and carbon nanotubes for tracking human movement.[29] Such materials, however, can require expensive starting materials and complex processing. An alternative approach using readily available materials would benefit the field. As a model application, we designed a system to translate the ASL alphabet because it requires a sophisticated integration of at least three types of sensors with electronics and data processing.

## Materials and methods

### Fabrication of piezoresistive strain sensors

A schematic diagram of the fabrication process is depicted in S2 Fig. Poly(dimethylsiloxane) (PDMS) (Dow Corning Slygard 184 with a base to cross-linker ratio of 20:1) was pour cast in a Petri dish and cured at 70°C for 1 h. To create the substrate, the PDMS was cut into strips with dimensions 3 cm × 0.5 cm × 340 μm. Carbon paint (Ted Pella DAG-T-502) was then painted on produce a piezoresistive film roughly 50 μm in thickness. Copper tape was then wrapped around each end of the sensor while a stainless steel thread was added to provide a secure electrical contact. Additional carbon paint was added on top of the device to reinforce the mechanical and electrical interface. Finally, the strain sensor was dipped in 10% polyurethane (PU) in tetrahydrofuran (THF) to provide an encapsulating layer.

### Characterization of strain sensors

Stress strain curves were measured using a Mark 10 pull tester and electrical measurements were performed with a Keithley 2400 sourcemeter.

### Finite element analysis

FEA models were produced in Autodesk Inventor, where the simulated sensor was deformed to the same radius of curvature as the wearable strain sensor (0.49 cm). The bottom edges of both sides of the sensor were constrained while 1.05 x 10^−3^ Pa were applied to the bottom face in an upward direction.

### Fabrication of the sensor glove

Nine piezoresistive sensors were fabricated and placed on the back of a leather athletic glove in locations corresponding with the metacarpal and proximal knuckles. Industrial strength, elastomeric adhesive E6000 (Eclectic Products, Inc) was used to bond the sensors to the glove. Conductive metal thread made from stainless steel was used to make the wearable circuits. E6000 was also used to adhere the thread and to insulate them from shorting with each other. Also using E6000, the custom designed PCB was adhered to the back of the leather glove on the Velcro strap to allow the wearer to easily put on and take off the glove.

### Designing the circuit board

The circuit board was designed in EAGLE CAD and board and circuit schematics are available in S3–S13 Figs. The PCB was designed to carry an on/off switch, a battery, power regulators, resistors, capacitors, inputs for the Teensy 3.1, the BLE nrf8001, the MPU 6050, and nine voltage divider circuits. The gerber files are available for download as S1 File.

### Code

The Teensy was coded in the Arduino IDE. This code is available for download as S2 File.

### Letter selection

Letters were selected by monitoring the state of each sensor, assigning a 0 or 1 depending on the amount the finger was bent (0 for relaxed, 1 for bent). The individual numbers for each knuckle were then concatenated into a nine-digit code by summing powers of 10 (see code). For example, if the hand were completely relaxed, the code would read “000000000” and if a fist were formed, bending all knuckles, the code would be “111111111.” Each letter was assigned a nine-digit key. The table used to determine which letter would be assigned which key is shown below in S14 Fig. This image shows the table, the order of the sensors in building the key, and which letters have degenerate keys along with which hardware would be required to differentiate between those letters. The outputs of the pressure sensor and accelerometer are shown in S15 Fig. A video of the gesture recognition and ASL translation is available for download as S3 File.

### Virtual hand

The glove sensor was interfaced with a virtual hand using a custom-built, object-oriented Python code. First, a twenty-four-node hand model was constructed in Cartesian coordinates. Each proximal and metacarpal knuckle was associated with three nodes to form a joint object. Each joint object had a bending state associated with it, as well as a method for adjusting the bending state using a standard geometrical transformation. A simple algorithm was designed to take in the voltage signals from the Arduino (either through a serial port, or a text file) and match the bending state of each knuckle on the virtual hand to that of the real hand. The trajectory of the hand was saved to a file for subsequent visualization with the open visualization tool OVITO[30]. The code is openly available on the following github repository: https://github.com/seroot/Glove. A video of the system recognizing gesture to control a virtual hand is available for download as S4 File.

## Results and discussion

**Fig 1:**
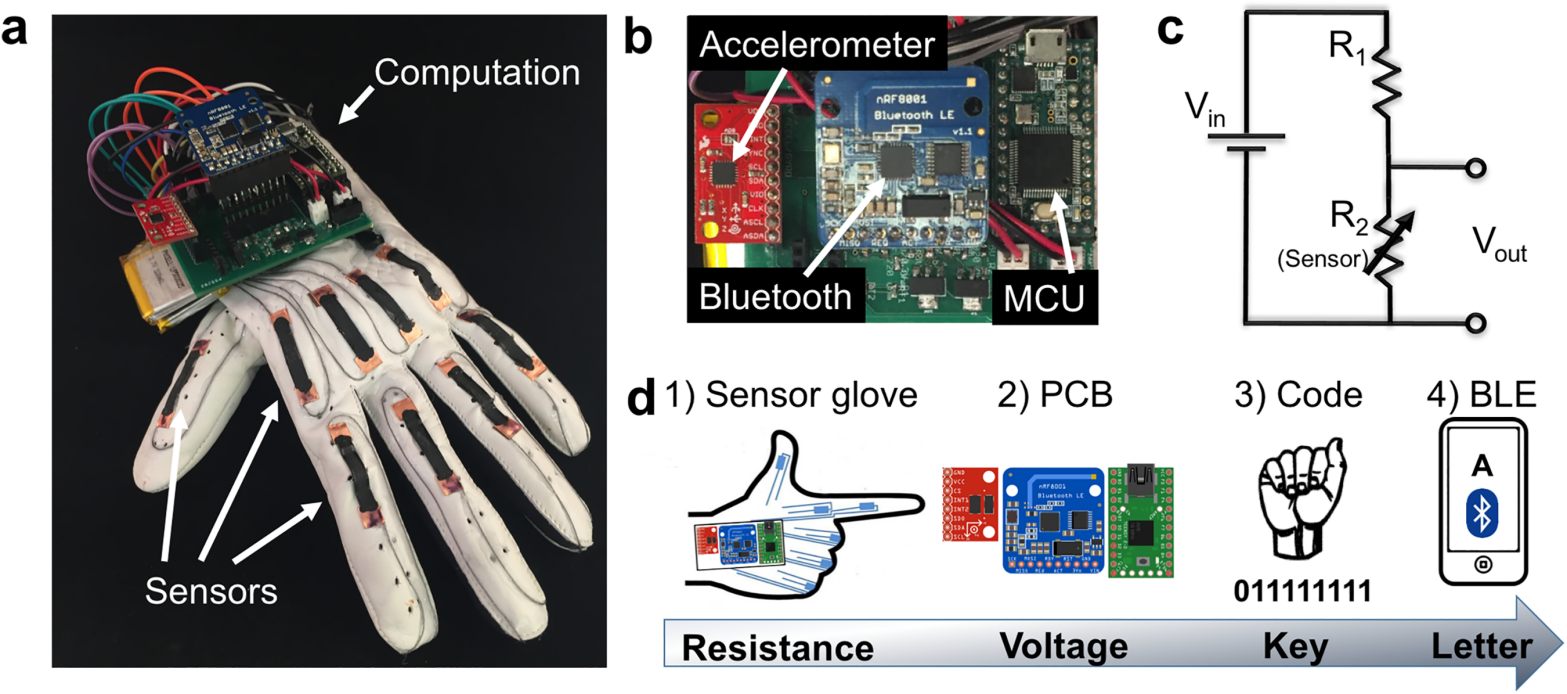
Overview of the gesture-decoding glove. (a) Photograph of the glove. (b) Photograph detailing the breakout boards for the MCU, accelerometer, and Bluetooth on the PCB. (c) A circuit diagram of a voltage divider. (d) Schematic drawing of wireless gesture recognition system and the flow of information. Step 1: a gesture was made and the strain sensors transduced the positions of the knuckles into variable resistance values. Step 2: the variable values of resistance were converted into voltages by the voltage dividers. Step 3: the MCU measured the nine voltages and, through a binary comparison process, used them to generate a nine-bit key. Step 4: the binary key was used to determine which letter was to be transmitted wirelessly.

The key aspects of the system are illustrated in Fig 1. The system prototype was constructed on the dorsal side of a leather athletic glove and comprised two main components: one for sensing, and one for computation (Fig 1A). The sensing component used nine elastomeric strain sensors, placed over each metacarpal and proximal knuckle, and one pressure sensor placed on the thumb. The computation component, detailed in Fig 1B, employed a custom printed circuit board (PCB) that integrated a microprocessor (Teensy 3.1), Bluetooth low-energy chip (BLE 4.0 nRF8001), and 6-axis accelerometer/gyroscope (MPU 6050) (Fig 1B). The PCB also incorporated nine voltage divider circuits (Fig 1C), which were used to convert the dynamically-varying resistance of each strain sensor into a voltage. Conductive thread was used to connect each sensor to the PCB. A schematic diagram of the flow of information is depicted in Fig 1D. The process began with a physical gesture, whereby the bending of different knuckles exerted different amounts of strain on each of the sensors. When the fingers were bent, the sensors underwent a reversible increase in their measured resistance. The complete process occurred in four steps: (1) a gesture was made and the strain sensors transduced the positions of the knuckles into variable resistance values; (2) the values of resistance were converted into voltages using the voltage dividers; (3) the Teensy microcontroller unit (MCU) measured each of the nine voltages and used them to generate a 9-bit binary key describing the state of each knuckle; and (4) the binary key was used to determine which letter was to be transmitted wirelessly. The computation was done onboard the wearable device to limit the amount of information that needed to be sent wirelessly and to keep the power consumption of the system low. An accelerometer and commercially available pressure sensor were added (S3 Fig) to enable the system to distinguish between letters with degenerate codes (E/S, G/L, H/K, R/U/V) or that required motion (I/J and D/Z). The glove was able to successfully determine all 26 letters of the alphabet (see Supporting Information for the criteria for letter detection).

**Fig 2:**
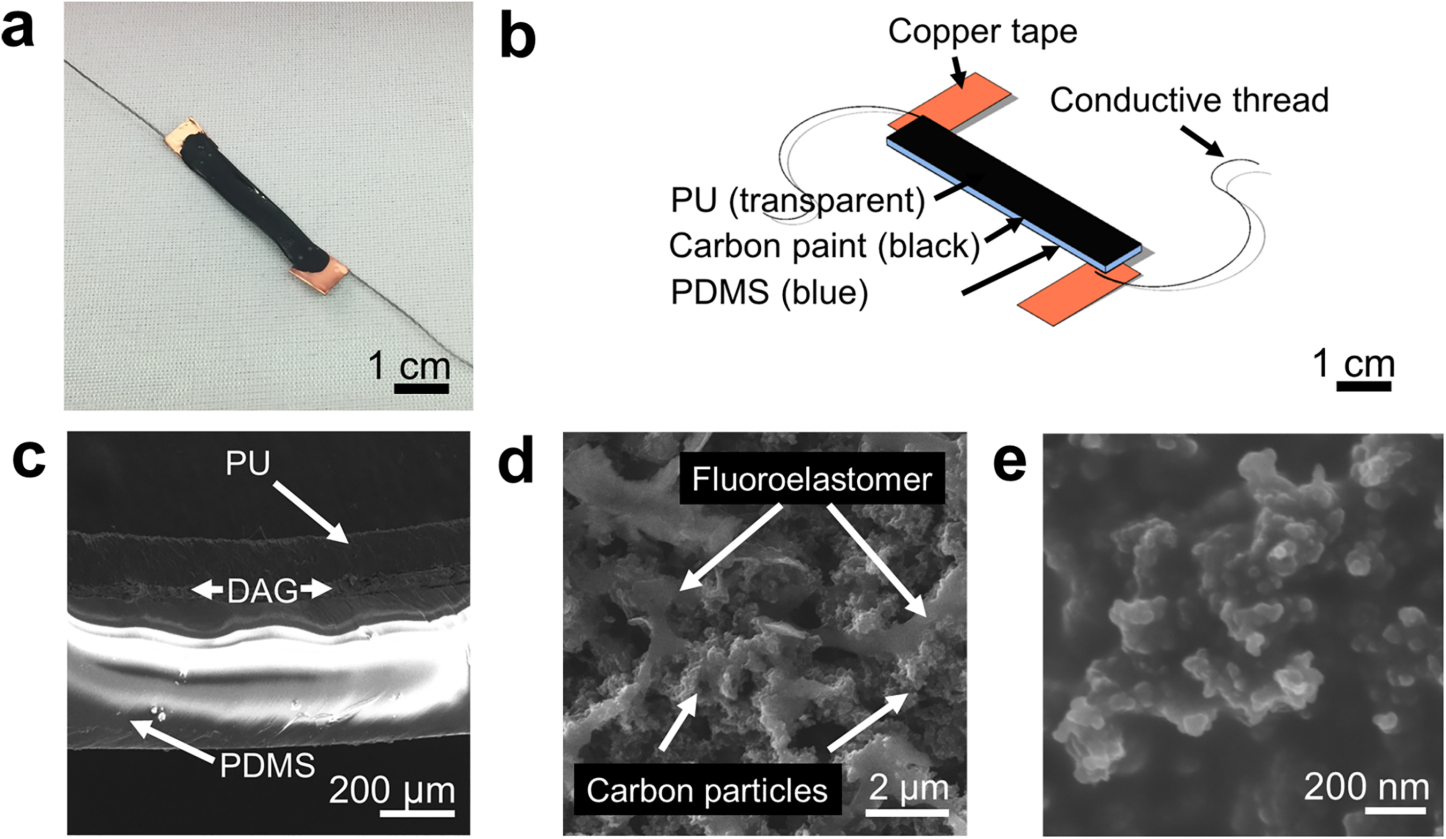
Overview of the wearable piezoresistive sensors. (a) Photograph of the sensor. (b) Schematic diagram of the sensor. (c) Cross-sectional optical micrograph of the sensor. (d) SEM image of the surface of the conductive fluoroelastomer. (e) SEM image of the nanoscopic carbon particles.

To transduce the bending motions of the knuckles into an electrical signal, mechanically compliant strain sensors were fabricated using a commercially available conductive composite (DAG-T-502, Ted Pella, Inc.) as the piezoresistive material (Fig 2A). Fig 2B illustrates the device layers in an exploded view. The sensors were fabricated by painting the composite layer (~50 μm) onto a PDMS substrate (~350 μm). Copper tape and stainless steel thread were then added before encapsulating the device in polyurethane (PU, ~200 μm). The average total thickness of the devices was 616 μm ± 50.2 μm. The PU encapsulant increased the durability of the sensor against abrasion, reinforced the weak interface between the copper tape and carbon ink, and prevented the delamination of the piezoresistive layer and the PDMS substrate after repeated cycles of deformation. A scanning electron microscope (SEM) image of the cross-sectional interface of the devices is shown in Fig 2C. Further magnification of the composite films (Fig 2D and Fig 2E) shows that the piezoresistive layer consists of nanoscale carbon particles (~100 nm) connected by an amorphous, elastomeric network. The sensors were designed to be low-cost and easy to process, have an easily measureable change in resistance (Δ*R*) when bent on the knuckles, and to output a consistent electrical signal over many strain cycles without interfering with the movement of the glove.

Materials for the piezoresistive sensor were selected because of their mechanical durability, ease of processing, low cost and commercial availability. The DAG-T-502 was mechanically stable enough to accommodate the interface between the stretchable conducting material and the copper contacts. PDMS substrates were simple to cast as smooth, thin substrates and the PU encapsulant provided increased mechanical stability using a simple dip coating method. Both the PDMS substrate (20:1 base:crosslinker) and PU encapsulant have low tensile moduli, 1 MPa[31] and 7.5 MPa.[32] Conductive thread was used instead of metal wiring to better integrate the circuitry with textiles. Open-source electronics were chosen for their low cost, availability, and ease of integrating the whole system. The goal was thus to make rapid prototyping and testing of new materials and hybrid electronic devices a realistic process for research laboratories who may not be experts in electrical engineering.

**Fig 3:**
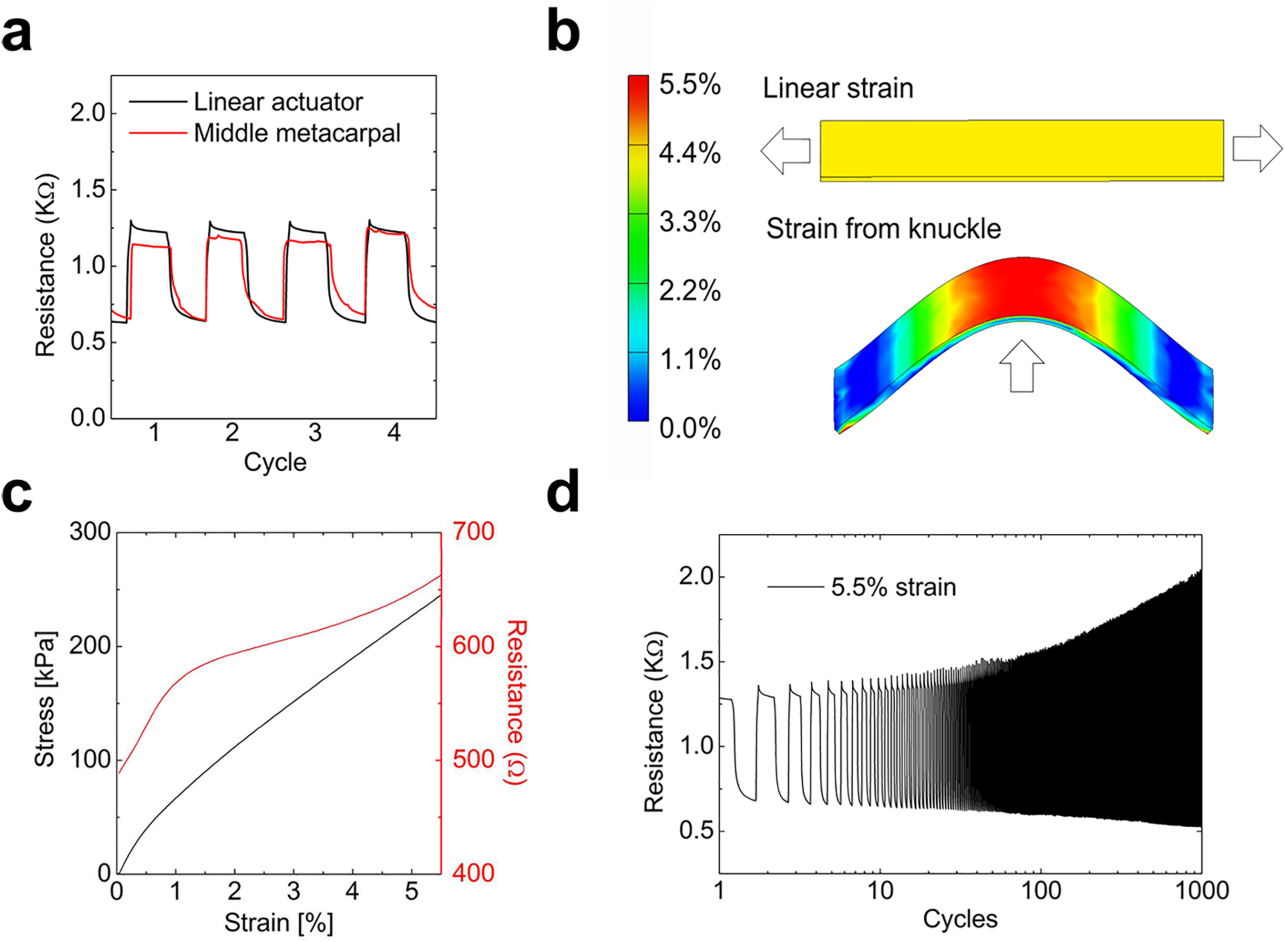
Characterization of the piezoresistance and FEA strain modeling of the sensors. (a) Resistance vs. strain of a representative strain sensor on the hand (red) and under controlled stretching conditions on a linear actuator (black). (b) Finite-element model simulating the strain distribution across the surface of the sensor in a linear stretching mode (top) and a knuckle strain regime (bottom). (c) Stress-strain (black) and resistance-strain (red) curves of the sensors measuring a tensile modulus of 3.9 MPa. (d) Resistance vs. strain of the same sample when cycled up to 1,000 times at 5.5%.

To investigate the strain distribution and fatigue experienced by the sensors during bending, four sensors were selected for electromechanical characterization. The sensors were adhered to the middle metacarpal position of a glove and a fist was tightly formed. The induced deformation and strain produced an average increase in resistance from *R*_unbent_ 560 ± 120 Ω to *R*_bent_ 1120 ± 280 Ω, corresponding an increase of resistance of a factor of 2 (Fig 3A, red curve). (Variation between sensors was attributed to the hand painting of the piezoelectric carbon layer and the distribution of resistance values can easily be accounted for in the calibration of the sensor system.) The sensor was then removed from the glove and placed on a linear actuator where controlled amounts of strain were used to achieve the same increase in resistance (Fig 3A, black curve). From this measurement, an average strain of 4.5 ± 1% was estimated across the sensor when the strain was applied linearly (Fig 3B top). Using FEA modeling, the strain distribution of a sensor under linear strain and of a sensor that was bent to the same radius of curvature as a knuckle-mounted sensor were simulated **(**Fig 3B bottom). From the FEA model of the bent sensor, the peak strain was estimated to be around 5.5%.

**Fig 4:**
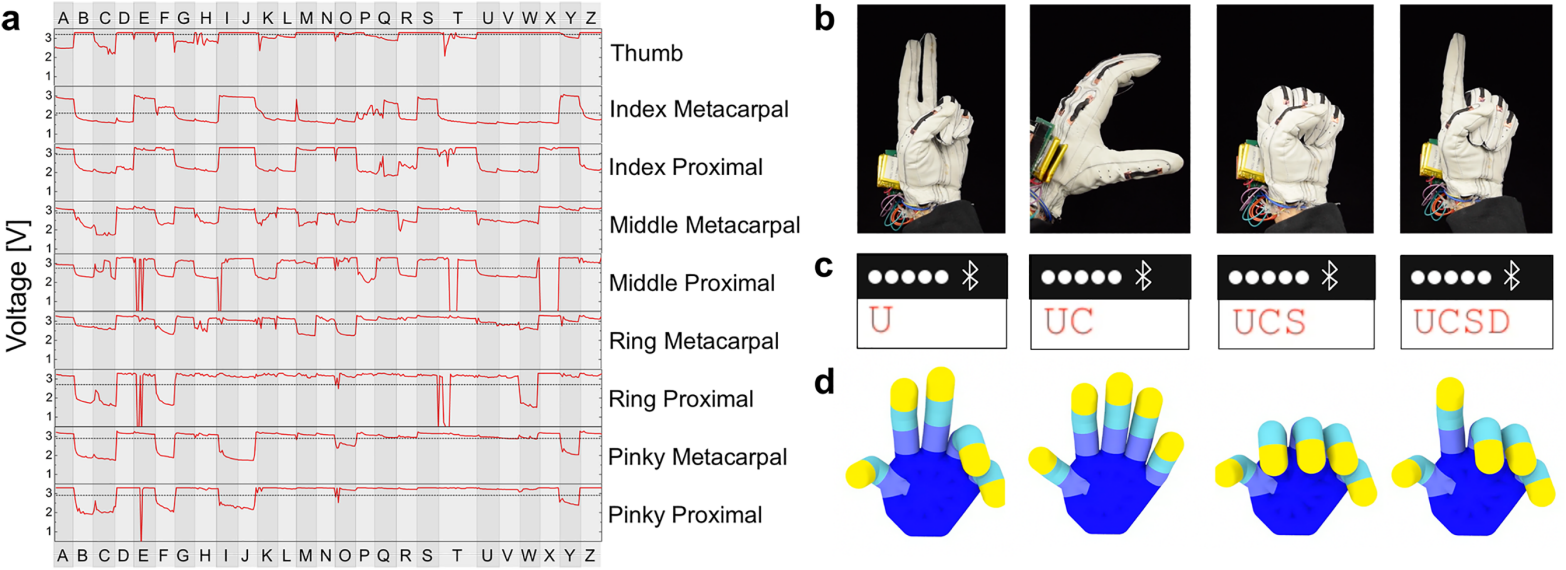
Deciphering hand gestures into the letters of ASL alphabet. (a) Voltage values associated with each knuckle while as the hand signs the letters of the ASL alphabet in order. Each sign was given ~30 s to form and hold the letter and, with the use of a pressure sensor and accelerometer, all 26 letters were successfully transcribed. The dotted lines represent the threshold value to determine the on/off state of the sensor. (b) Photographs of the hand in configurations corresponding to the ASL gestures for the letters ‘U’ ‘C’ ‘S’ and ‘D’. (c) Screen shots of a smartphone as the letters were received to construct a word. (d) Images of a virtual hand in which the bending state of each finger corresponded to the resistance values of the sensors.

Fig 3C (red) shows the experimentally measured non-linear resistance as a function of strain. Analysis of the stress-strain curve (Fig 3C, black curve) revealed a tensile modulus of 3.9 MPa and elastic behavior within the working range of the sensors. Though the modulus is still roughly 100 times that of skin,[33] the sensors are not perceptible by the user the through the glove, which is much stiffer than the sensors. Sensors had a near instantaneous response time when strained, measured to be 0.2 ± 0.05 s and a significantly larger response time related to the relaxation of the fingers, measured to be 1.7 ± 0.1 s, due to viscoelastic properties of the stretchable sensor. A plot of the hysteresis can be found in the Supporting Information (S16 Fig) Finally, the elastomeric sensors were repeatedly strained to their peak strain to determine the effect of fatigue on the electrical signal (Fig 4D). After 1,000 cycles, the relative change in resistance increased from 1.2 to 2.9, but the ability of the system to determine the correct letter was preserved, as the code responsible for letter detection is only dependent on the resistance exceeding a preset threshold voltage. In fact, a larger increase in resistance with strain potentially improve the ability to detect the letter, as it is the differences in resistance that matter.

The MCU was programmed to determine the correct letter by generating a nine-digit binary key based on the on/off states of the nine sensors. The red curves in Fig 4A show the voltage values associated with each sensor as the hand signed the letters of the ASL alphabet in order. When the knuckle was bent, the value of resistance across the sensor (and thus the value of the voltage measured by the MCU) increased. If the value of the voltage was measured to be higher than that of the pre-programmed threshold value (dotted horizontal line in each chart), the sensor was assigned a 1. A knuckle not sufficiently bent was assigned a 0. A nine-digit key was then formed by concatenating the individual values assigned to each sensor, and these keys were associated with corresponding letters of the ASL alphabet (they key generation table is available in S14 Fig). Fig 4B shows the hand in configurations corresponding to the letters ‘U’ ‘C’ ‘S’ and ‘D’. These letters were sent wirelessly to a smartphone (Fig 4C and video S3 File). By feeding the serial output of the sensors into a model of a virtual hand, we were able to make a virtual hand reproduce the ASL sign gestures (Fig 4D and video S4 File**)**. Using an accelerometer and pressure sensor to enable the system to distinguish between letters with degenerate codes (E/S, G/L, H/K, R/U/V) or that required motion (I/J and D/Z), the glove was able to determine all 26 letters of the alphabet (S15 Fig).

## Conclusion

Through the integration of piezoresistive elastomers, open-source computation, and low-energy Bluetooth, we developed a low-cost system for decoding and transmitting human hand gestures. This system can serve as a test-bed platform for new materials, flexible hybrid electronics, and low-power circuits in human-machine interfaces. Attractive features of the system are low-cost (less than $100), modularity (the materials and components can be exchanged), and a complete description (in the Supporting Information), which will allow other laboratories to use the system. In particular, the stretchable conductive elastomer used as the piezoresistive sensors is commercially available and thus may play a similar role in stretchable electronics for human-machine interfaces as PDMS now plays in micropatterning and soft robotics. While the electronic components used to decode and transmit the data are modified from rigid, off-the-shelf components, there is an opportunity to make purpose-designed components in flexible, hybrid form factors. The materials and methods described here for the recognition of human gestures could also be applied to gather biometric and diagnostic data. The availability of open-sourced, test-bed systems can accelerate the development of materials and integration strategies for low-cost human-machine interfaces.

## Supporting information

S1 Fig Parts list and cost. Cost of parts to fabricate the sensor glove. Adjusted costs were estimated at $1.00 for cases in which a very small amount of the contents of the container indicated were used.
(TIF)

S2 Fig Fabrication schematic of the piezoresistive sensors. A schematic diagram of the fabrication process is depicted in S1 Fig. Poly(dimethylsiloxane) (PDMS) (Dow Corning Slygard 184 with a base to cross-linker ratio of 20:1) was pour cast in a Petri dish and cured at 70°C for 1 h. To create the substrate, the PDMS was cut into strips with dimensions 3 cm × 0.5 cm × 340 μm. Carbon paint (Ted Pella DAG-T-502) was then painted on produce a piezoresistive film roughly 50 μm in thickness. Copper tape was then wrapped around each end of the sensor while a stainless steel thread was added to provide a secure electrical contact. Additional carbon paint was added on top of the device to reinforce the mechanical and electrical interface. Finally, the strain sensor was dipped in 10% polyurethane (PU) in tetrahydrofuran (THF) to provide an encapsulating layer.
(TIF)

S3 Fig Designing the circuit board. Image of the EAGLE CAD board. The PCB was designed to carry an on/off switch, a battery, power regulators, resistors, capacitors, inputs for the Teensy 3.1, the BLE nrf8001, the MPU 6050, and nine voltage divider circuits. The gerber files (“gerber.zip”) are attached to the SI and the PCB board was fabricated at 4pcb.com.
(TIF)

S4 Fig EAGLE CAD circuit schematic for the power system. Image of the EAGLE CAD circuit schematic for the power system.
(TIF)

S5 Fig EAGLE CAD circuit schematic for the microcontroller. Image of the EAGLE CAD circuit schematic for the microcontroller.
(TIF)

S6 Fig EAGLE CAD circuit schematic for the Bluetooth system. Image of the EAGLE CAD circuit schematic for the Bluetooth system.
(TIF)

S7 Fig EAGLE CAD circuit schematic for the thumb sensor. Image of the EAGLE CAD circuit schematic for the thumb sensor.
(TIF)

S8 Fig EAGLE CAD circuit schematic for the index sensors. Image of the EAGLE CAD circuit schematic for the index sensors.
(TIF)

S9 Fig EAGLE CAD circuit schematic for the middle finger sensors. Image of the EAGLE CAD circuit schematic for the middle finger sensors.
(TIF)

S10 Fig EAGLE CAD circuit schematic for the ring finger sensors. Image of the EAGLE CAD circuit schematic for the ring finger sensors.
(TIF)

S11 Fig EAGLE CAD circuit schematic for the pinky sensor system. Image of the EAGLE CAD circuit schematic for the pinky sensor system.
(TIF)

S12 Fig EAGLE CAD circuit schematic for the touch sensor. Image of the EAGLE CAD circuit schematic for the touch sensor. The system was designed with two touch sensors but only one was needed.
(TIF)

S13 Fig EAGLE CAD circuit schematic for the accelerometer/gyroscope. Image of the EAGLE CAD circuit schematic for the accelerometer/gyroscope.
(TIF)

S14 Fig Letter selection table. Key generation table indicating which letters correspond to which keys. The table also shows which letters have redundant keys and which type of hardware was used to differentiate those redundant letters. Letters were selected by monitoring the state of each sensor, assigning a 0 or 1 depending on the amount the finger was bent (0 for relaxed, 1 for bent). The individual numbers for each knuckle were then concatenated into a nine-digit code by summing powers of 10 (see code). For example, if the hand were completely relaxed, the code would read “000000000” and if a fist were formed, bending all knuckles, the code would be “111111111.” Each letter was assigned a nine-digit key. The table used to determine which letter would be assigned which key is shown below in S5 Fig. This image shows the table, the order of the sensors in building the key, and which letters have degenerate keys along with which hardware would be required to differentiate between those letters.
(TIF)

S15 Fig Determining degenerate keys using pressure sensors and an accelerometer. Table depicting the serial outputs of the parameters used to differentiate between letters with degenerate keys. A pressure sensor was used to differentiate between E/S, G/L, H/K, and R/U/V while an accelerometer was used to decouple the letters D/Z and I/J. The threshold for the x-acceleration was set to |3000|, while the pressure sensor was set to Pressure = 3.3 for high, 3.3 > Pressure ≥ 1.0 for med, and 1.0 > Pressure for low.
(TIF)

S16 Fig Hysteresis of the stretchable strain sensors. Hysteresis of the resistance vs. strain as the sensor was stretched from rest to 5.5%, the controllably released back to its initial length. (The drop in resistance at 5.5% is due to the pause of the machine and viscoelastic effects in the sensor).
(TIF)

S1 File PCB gerber files. (ZIP)

S2 File Arduino code. (INO)

S3 File Video of gesture recognition and ASL translation. (MP4)

S4 File Video of gesture recognition and control of the virtual hand. (MP4)
