# Supplementary figures and images for: The Language of Glove: Wireless gesture decoder with low-power and stretchable hybrid electronics
Source: PLoS One. 2017 Jul 12;12(7):e0179766. doi: 10.1371/journal.pone.0179766 (PMC5507487; doi:10.1371/journal.pone.0179766)

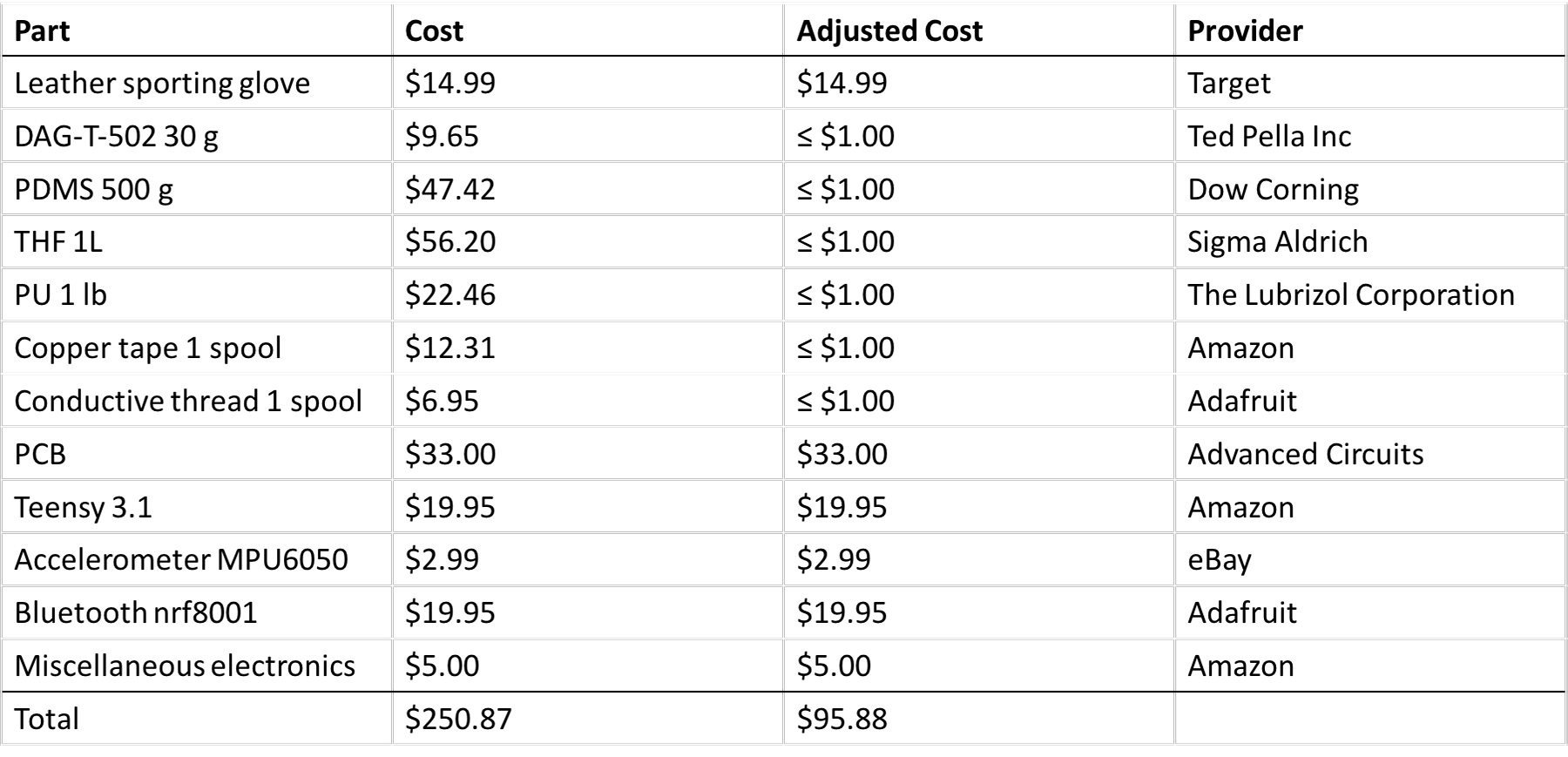

Supplement: S1 Fig — Cost of parts to fabricate the sensor glove. Adjusted costs were estimated at $1.00 for cases in which a very small amount of the contents of the container indicated were used. (TIF) [file pone.0179766.s001.tif]

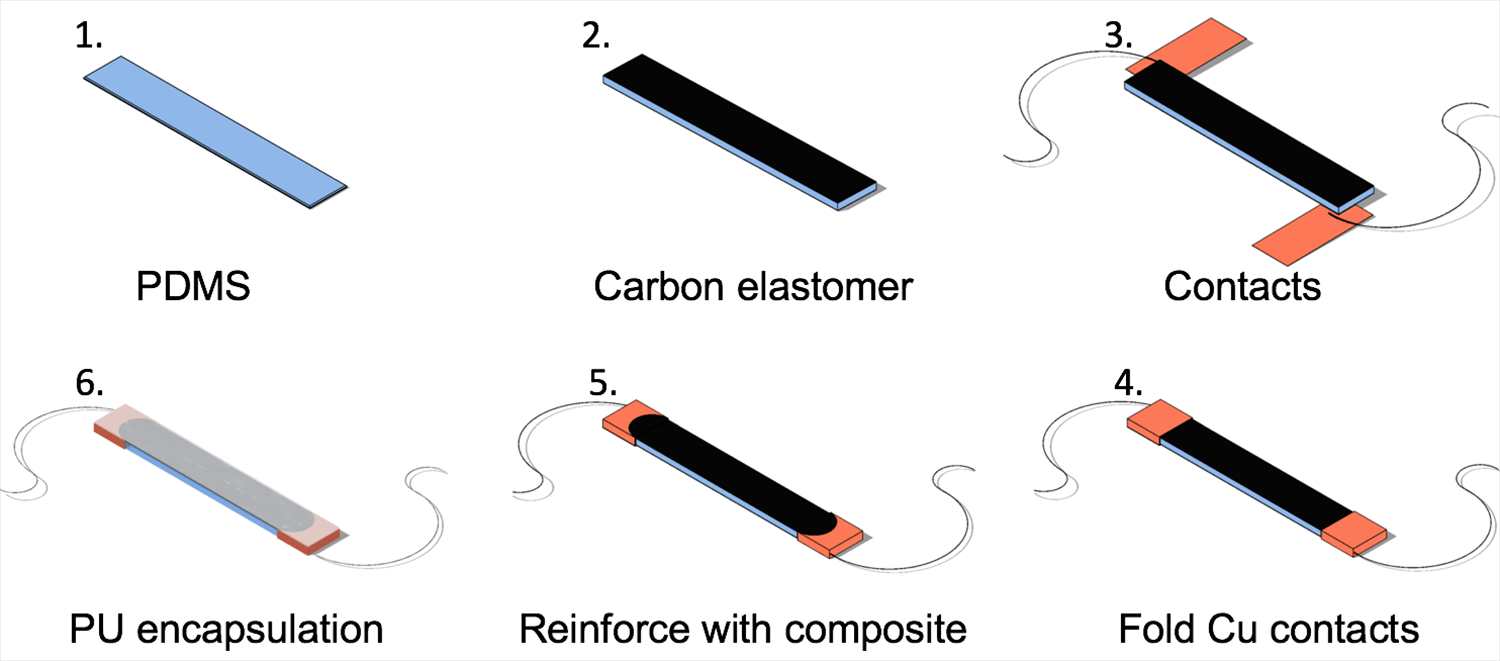

Supplement: S2 Fig — A schematic diagram of the fabrication process is depicted in S1 Fig. Poly(dimethylsiloxane) (PDMS) (Dow Corning Slygard 184 with a base to cross-linker ratio of 20:1) was pour cast in a Petri dish and cured at 70°C for 1 h. To create the substrate, the PDMS was cut into strips with dimensions 3 cm × 0.5 cm × 340 μm. Carbon paint (Ted Pella DAG-T-502) was then painted on produce a piezoresistive film roughly 50 μm in thickness. Copper tape was then wrapped around each end of the sensor while a stainless steel thread was added to provide a secure electrical contact. Additional carbon paint was added on top of the device to reinforce the mechanical and electrical interface. Finally, the strain sensor was dipped in 10% polyurethane (PU) in tetrahydrofuran (THF) to provide an encapsulating layer. (TIF) [file pone.0179766.s002.tif]

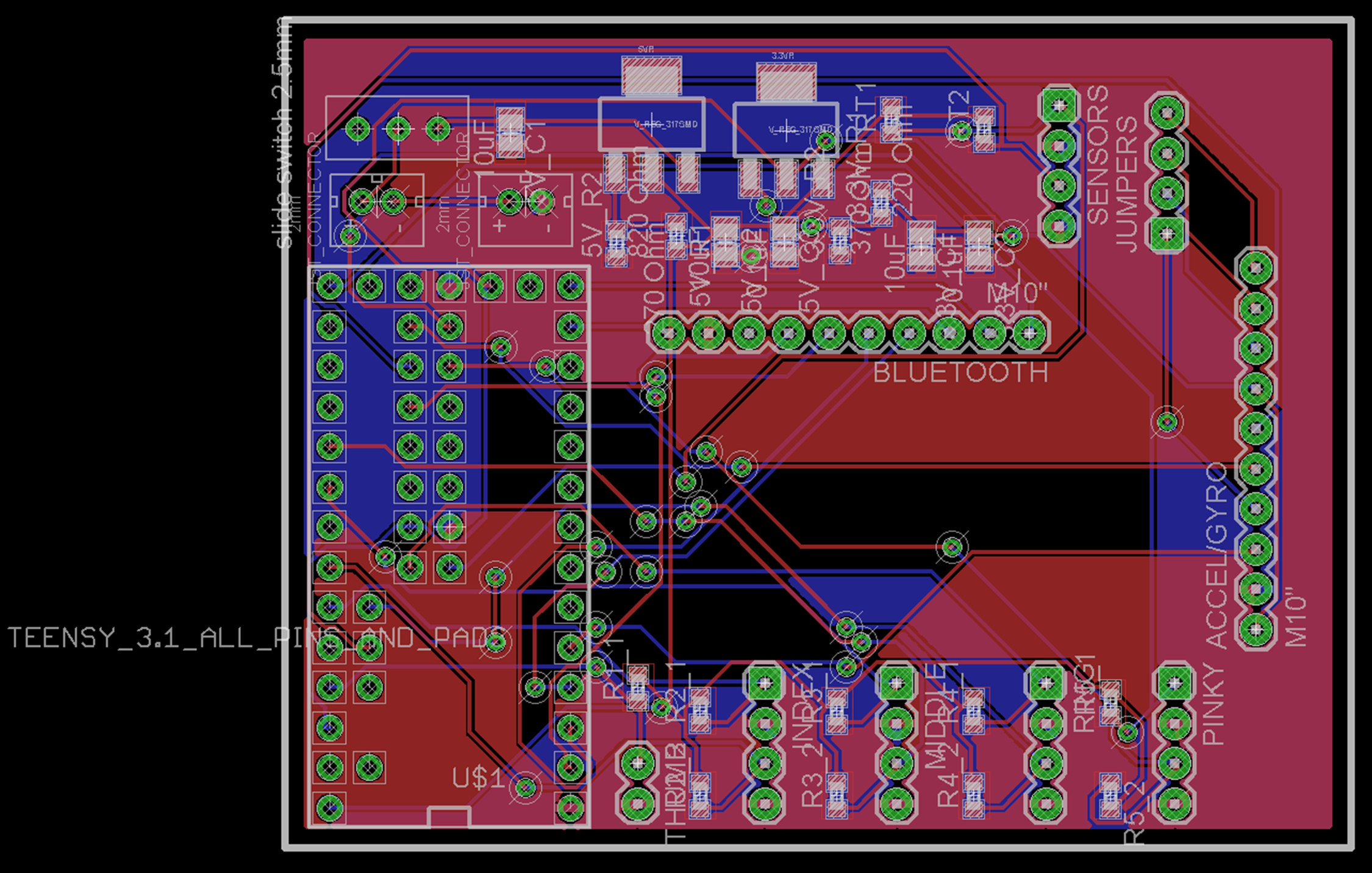

Supplement: S3 Fig — Image of the EAGLE CAD board. The PCB was designed to carry an on/off switch, a battery, power regulators, resistors, capacitors, inputs for the Teensy 3.1, the BLE nrf8001, the MPU 6050, and nine voltage divider circuits. The gerber files (“gerber.zip”) are attached to the SI and the PCB board was fabricated at 4pcb.com. (TIF) [file pone.0179766.s003.tif]

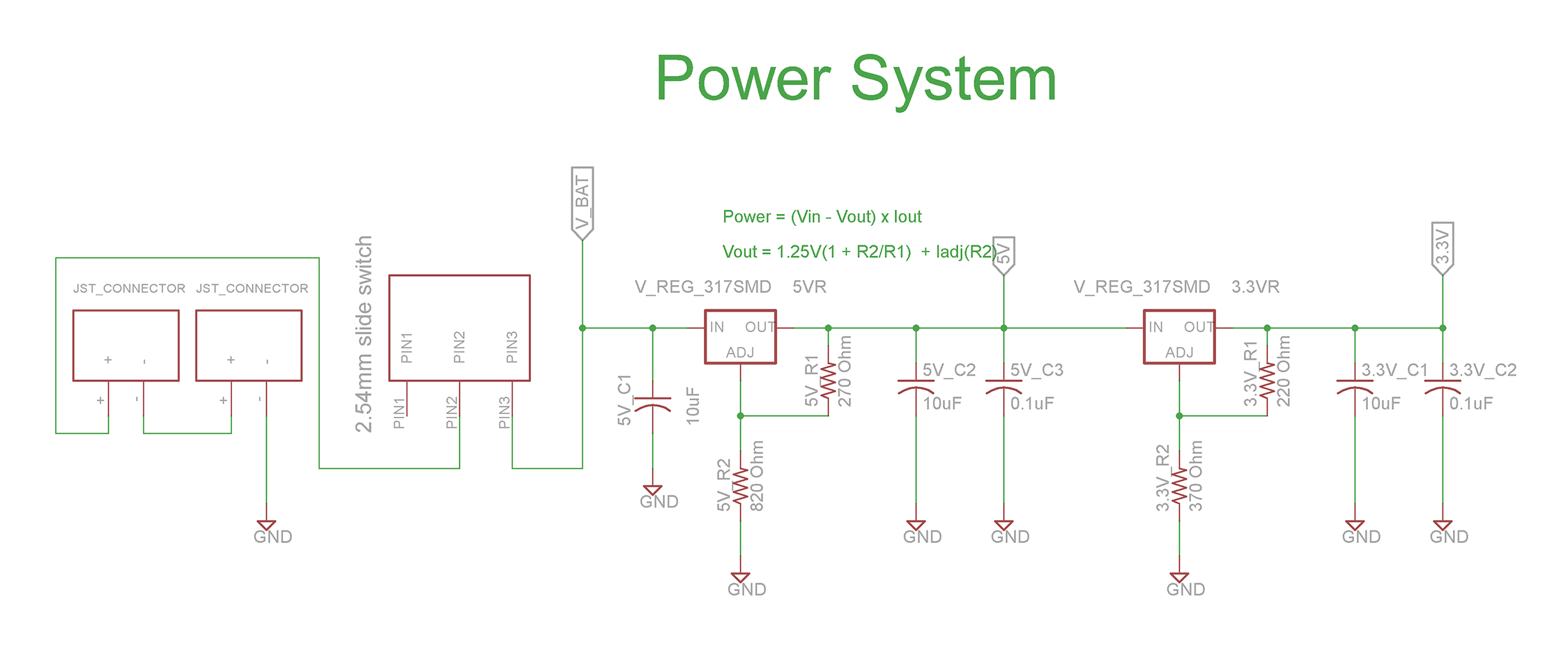

Supplement: S4 Fig — Image of the EAGLE CAD circuit schematic for the power system. (TIF) [file pone.0179766.s004.tif]

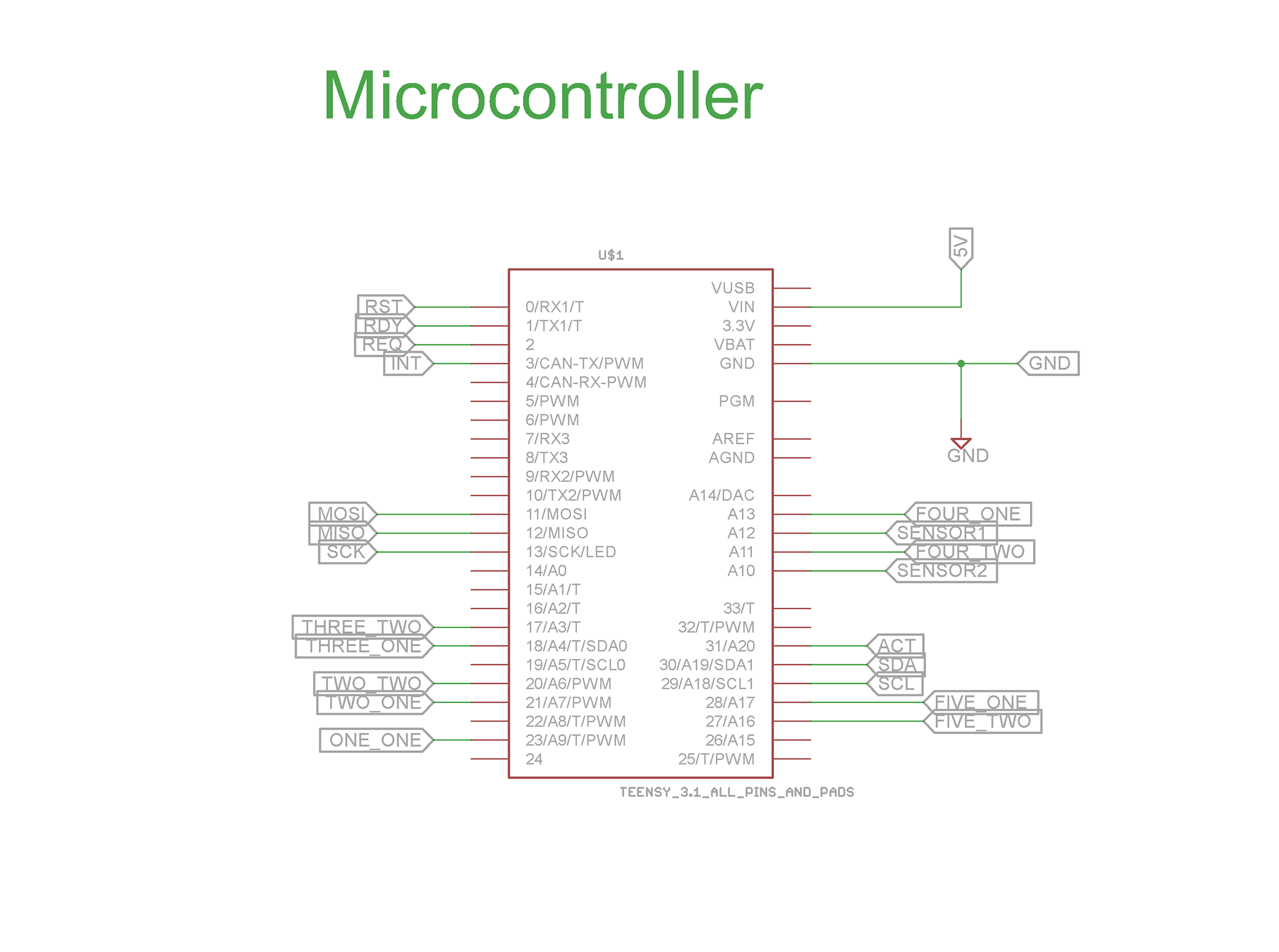

Supplement: S5 Fig — Image of the EAGLE CAD circuit schematic for the microcontroller. (TIF) [file pone.0179766.s005.tif]

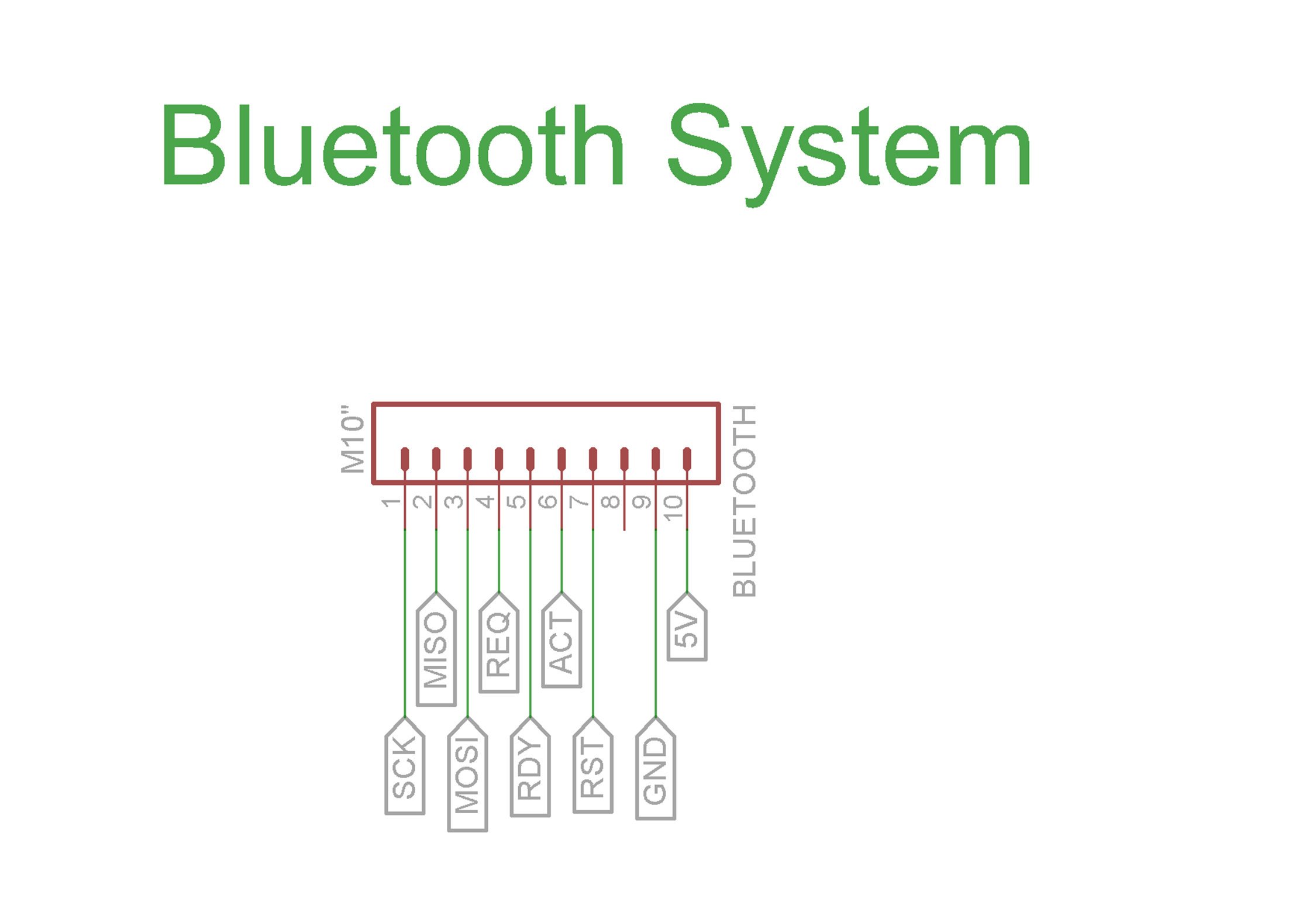

Supplement: S6 Fig — Image of the EAGLE CAD circuit schematic for the Bluetooth system. (TIF) [file pone.0179766.s006.tif]

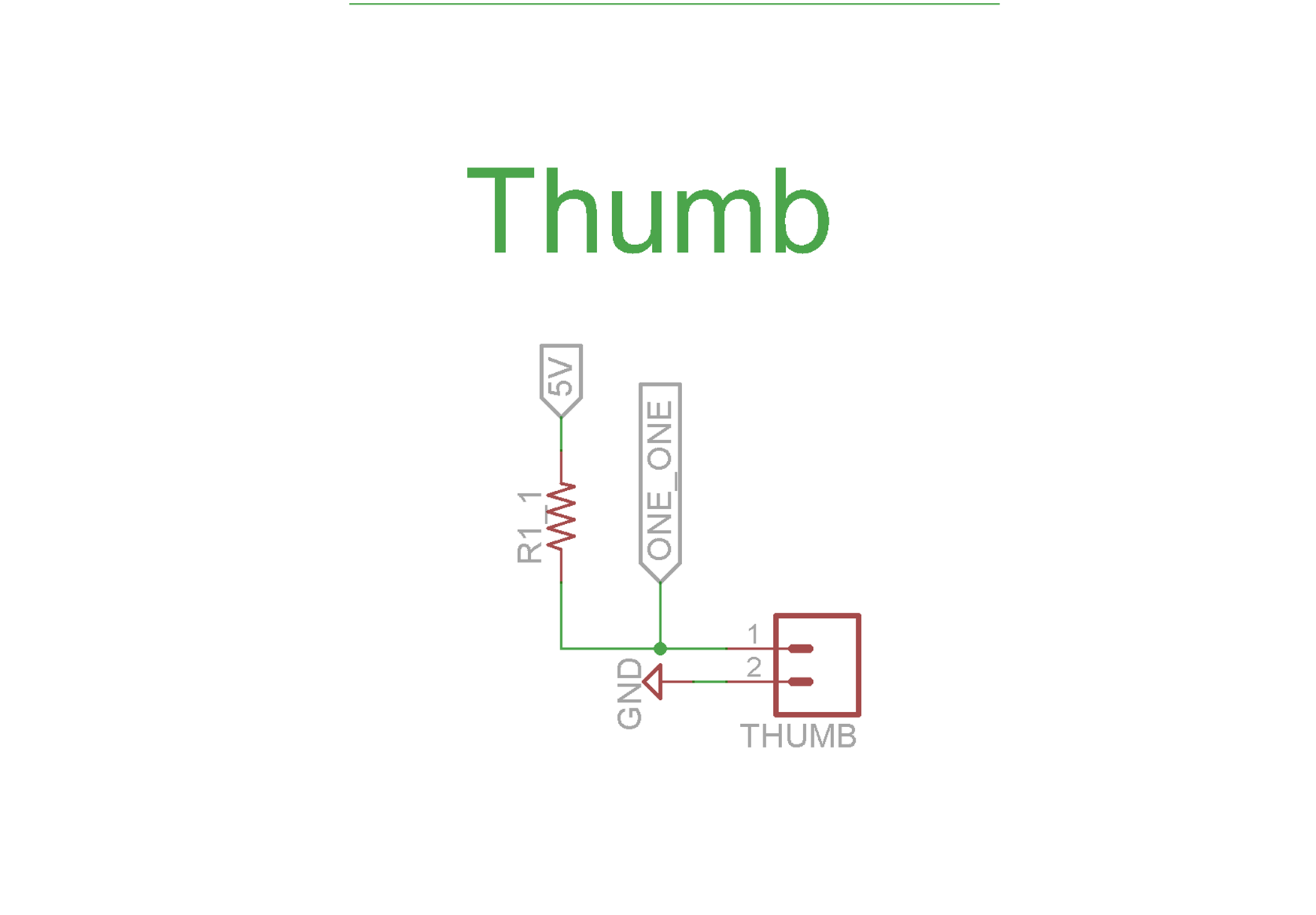

Supplement: S7 Fig — Image of the EAGLE CAD circuit schematic for the thumb sensor. (TIF) [file pone.0179766.s007.tif]

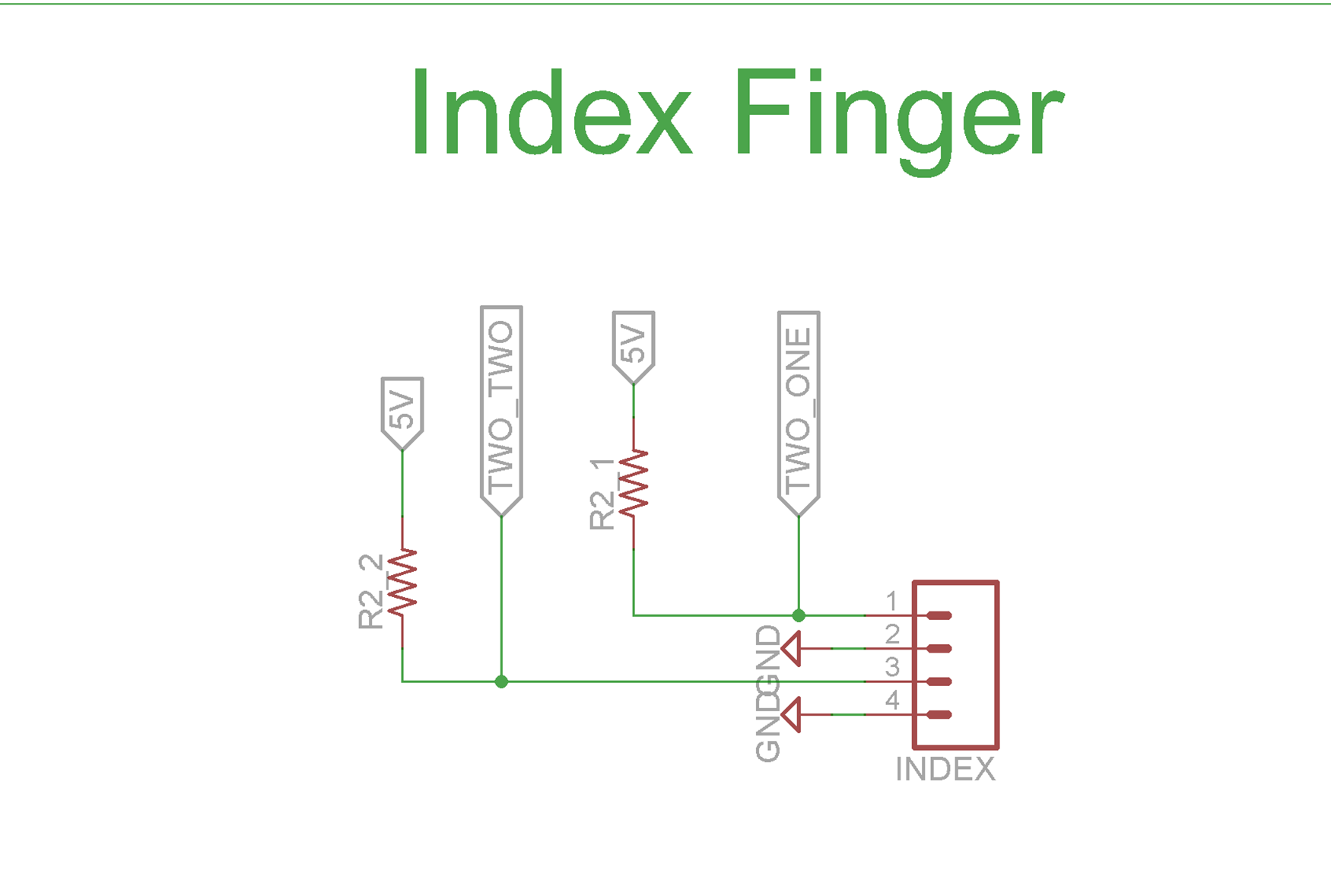

Supplement: S8 Fig — Image of the EAGLE CAD circuit schematic for the index sensors. (TIF) [file pone.0179766.s008.tif]

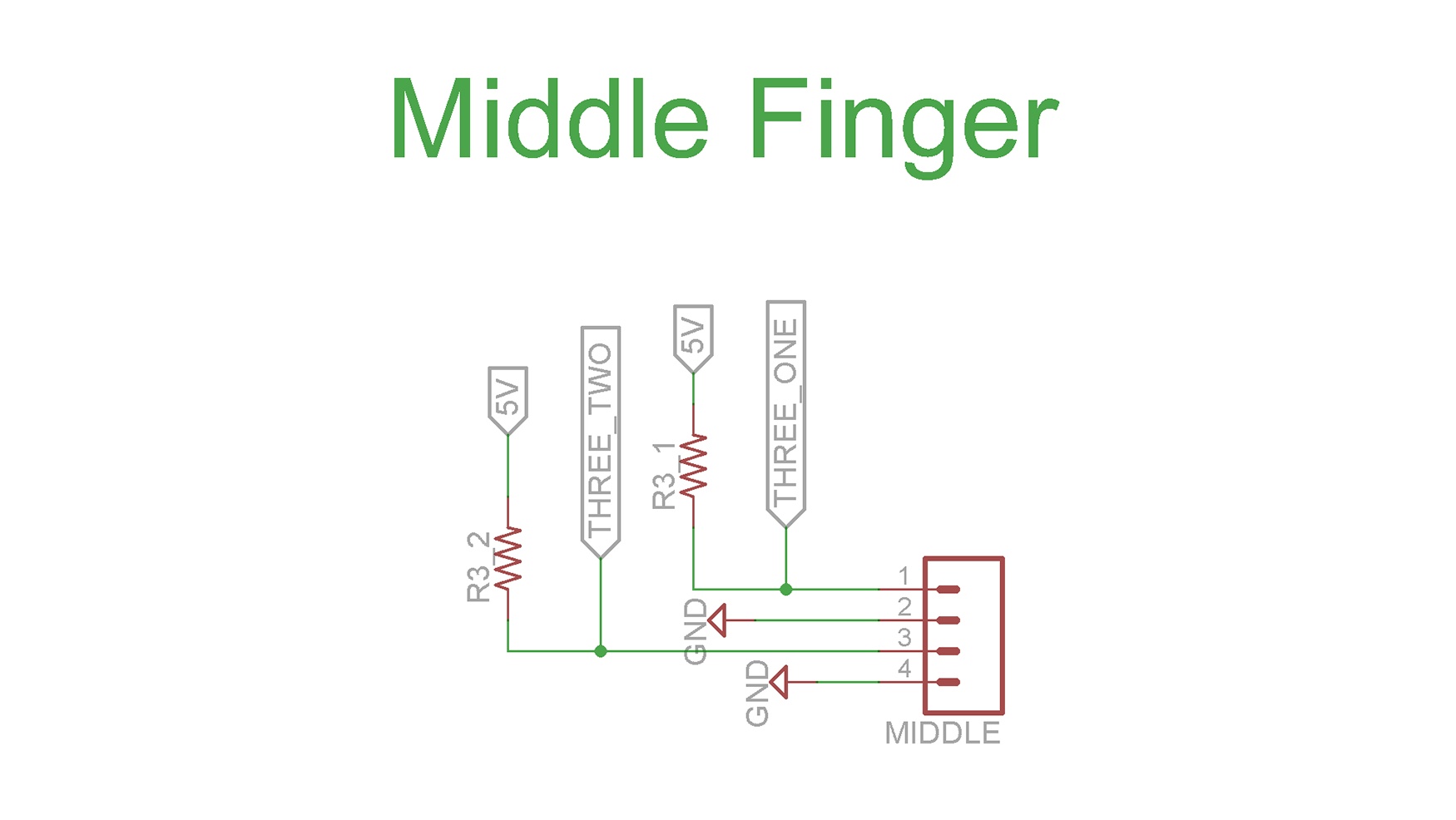

Supplement: S9 Fig — Image of the EAGLE CAD circuit schematic for the middle finger sensors. (TIF) [file pone.0179766.s009.tif]

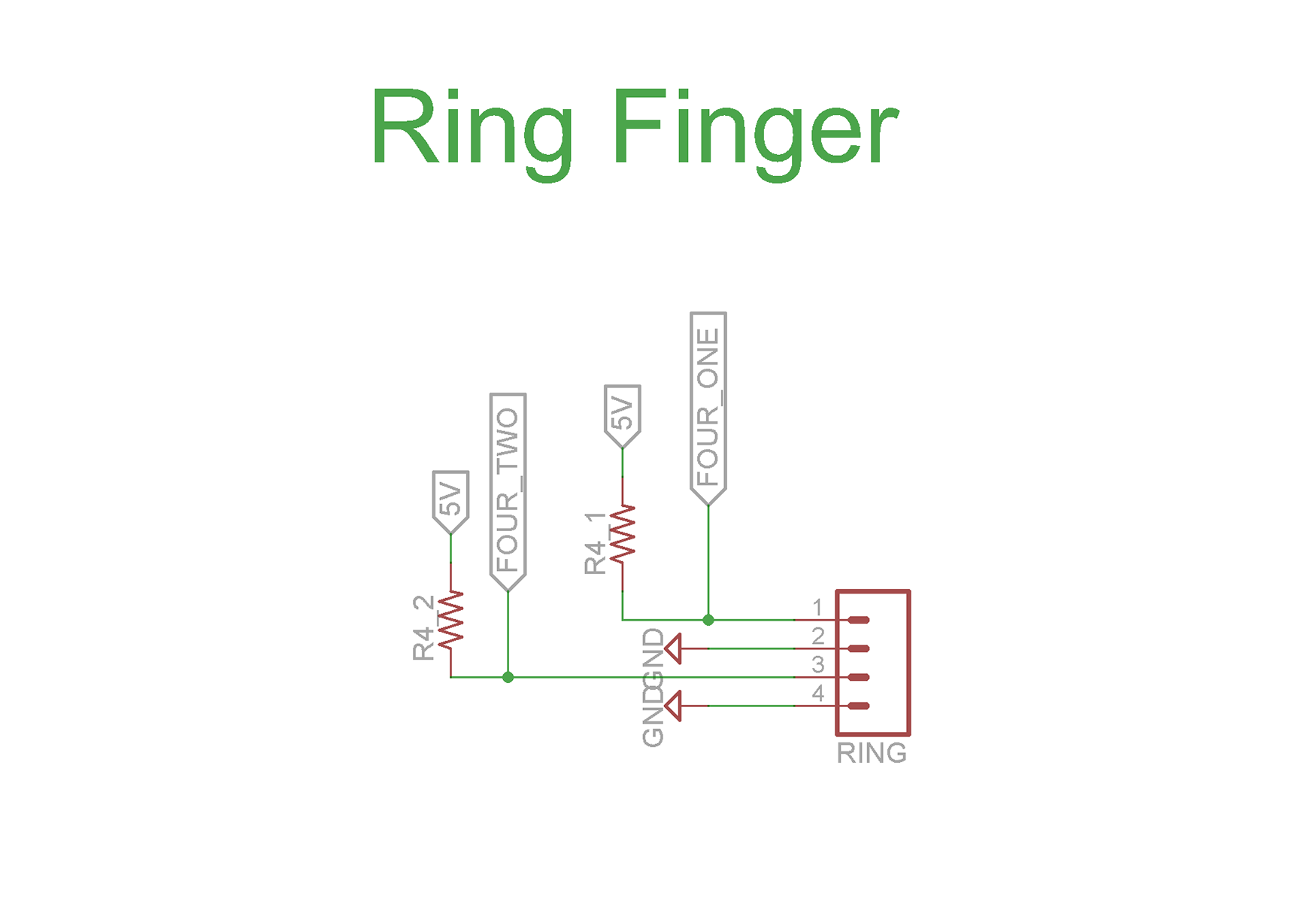

Supplement: S10 Fig — Image of the EAGLE CAD circuit schematic for the ring finger sensors. (TIF) [file pone.0179766.s010.tif]

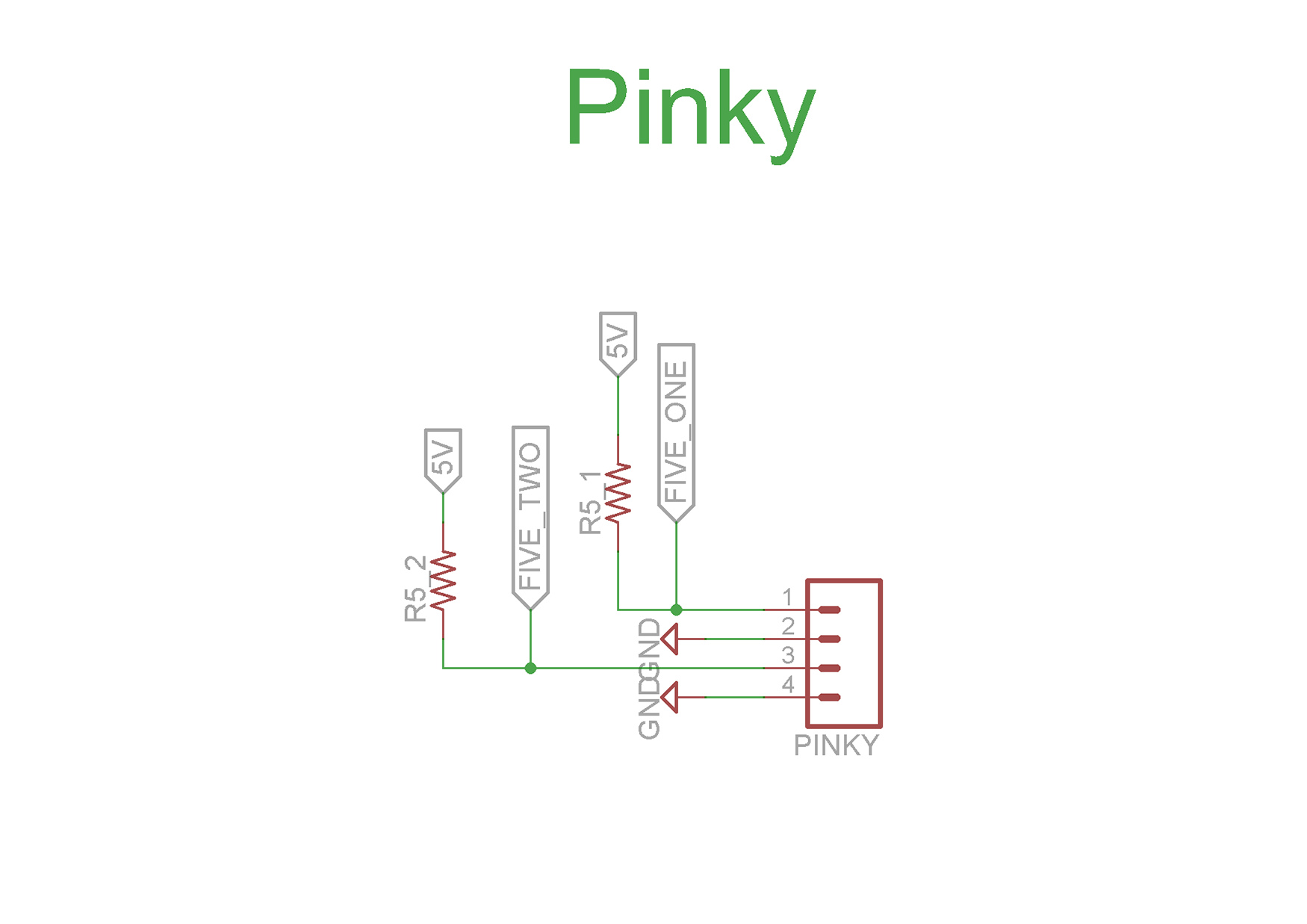

Supplement: S11 Fig — Image of the EAGLE CAD circuit schematic for the pinky sensor system. (TIF) [file pone.0179766.s011.tif]

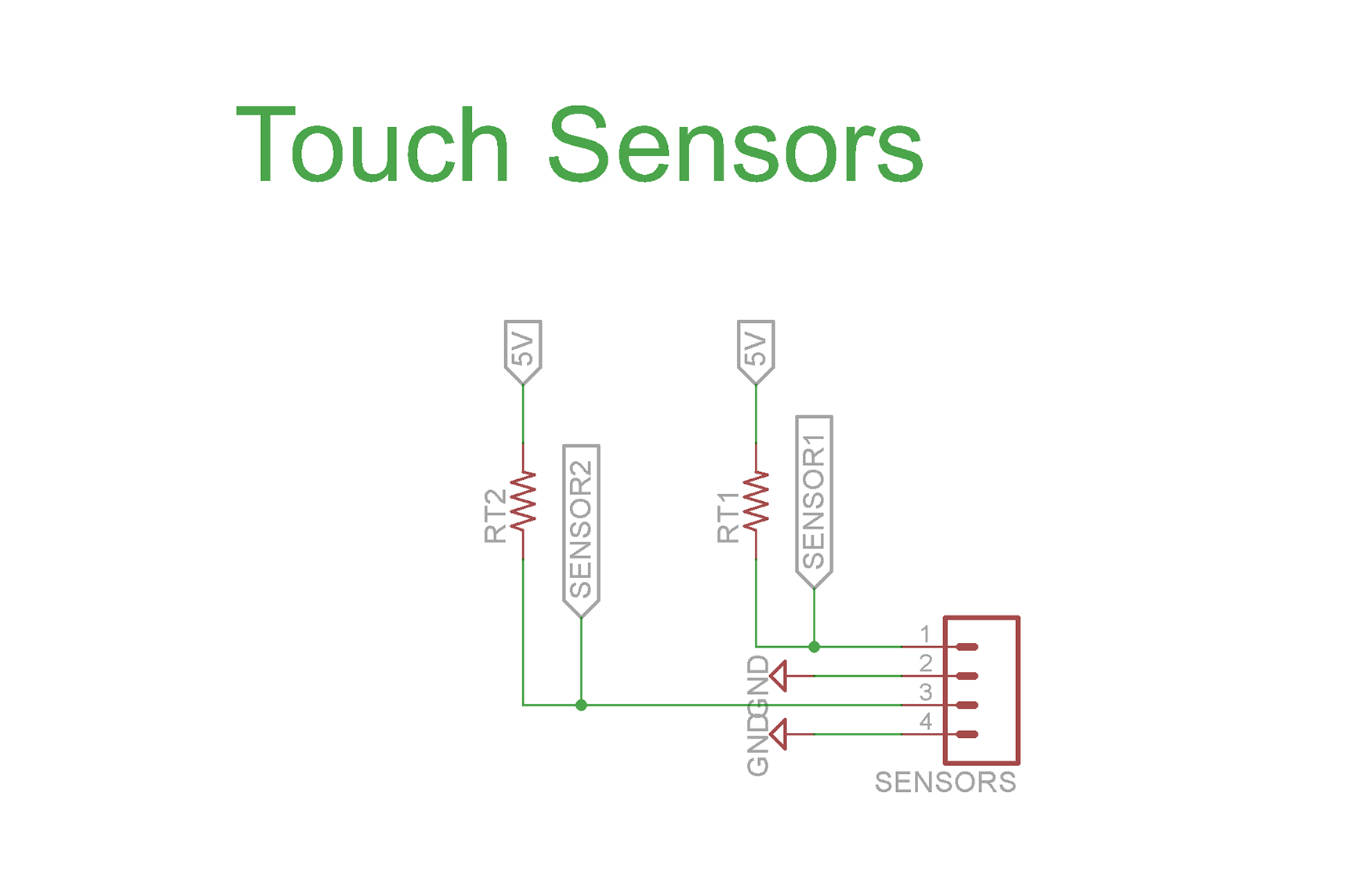

Supplement: S12 Fig — Image of the EAGLE CAD circuit schematic for the touch sensor. The system was designed with two touch sensors but only one was needed. (TIF) [file pone.0179766.s012.tif]

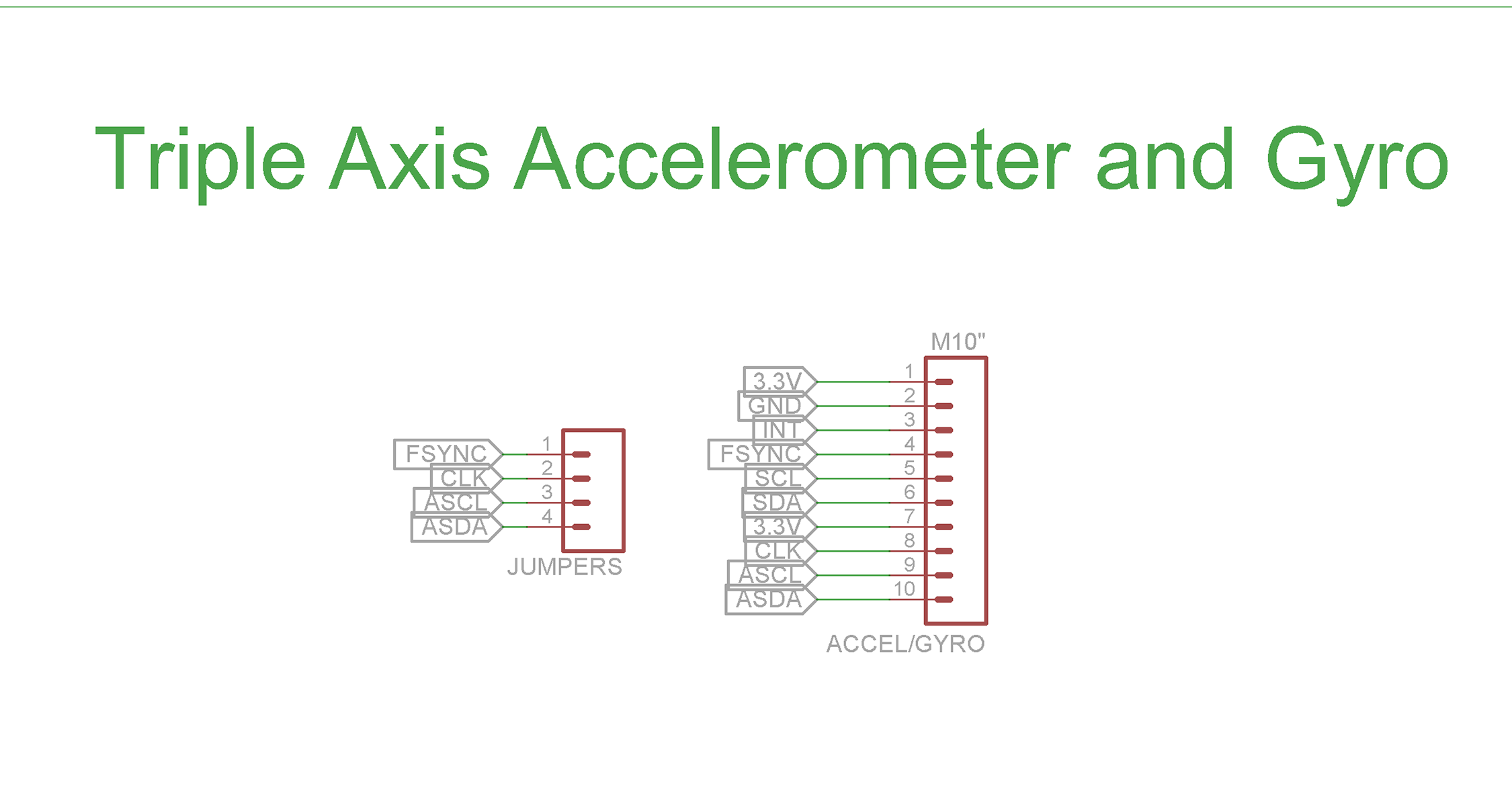

Supplement: S13 Fig — Image of the EAGLE CAD circuit schematic for the accelerometer/gyroscope. (TIF) [file pone.0179766.s013.tif]

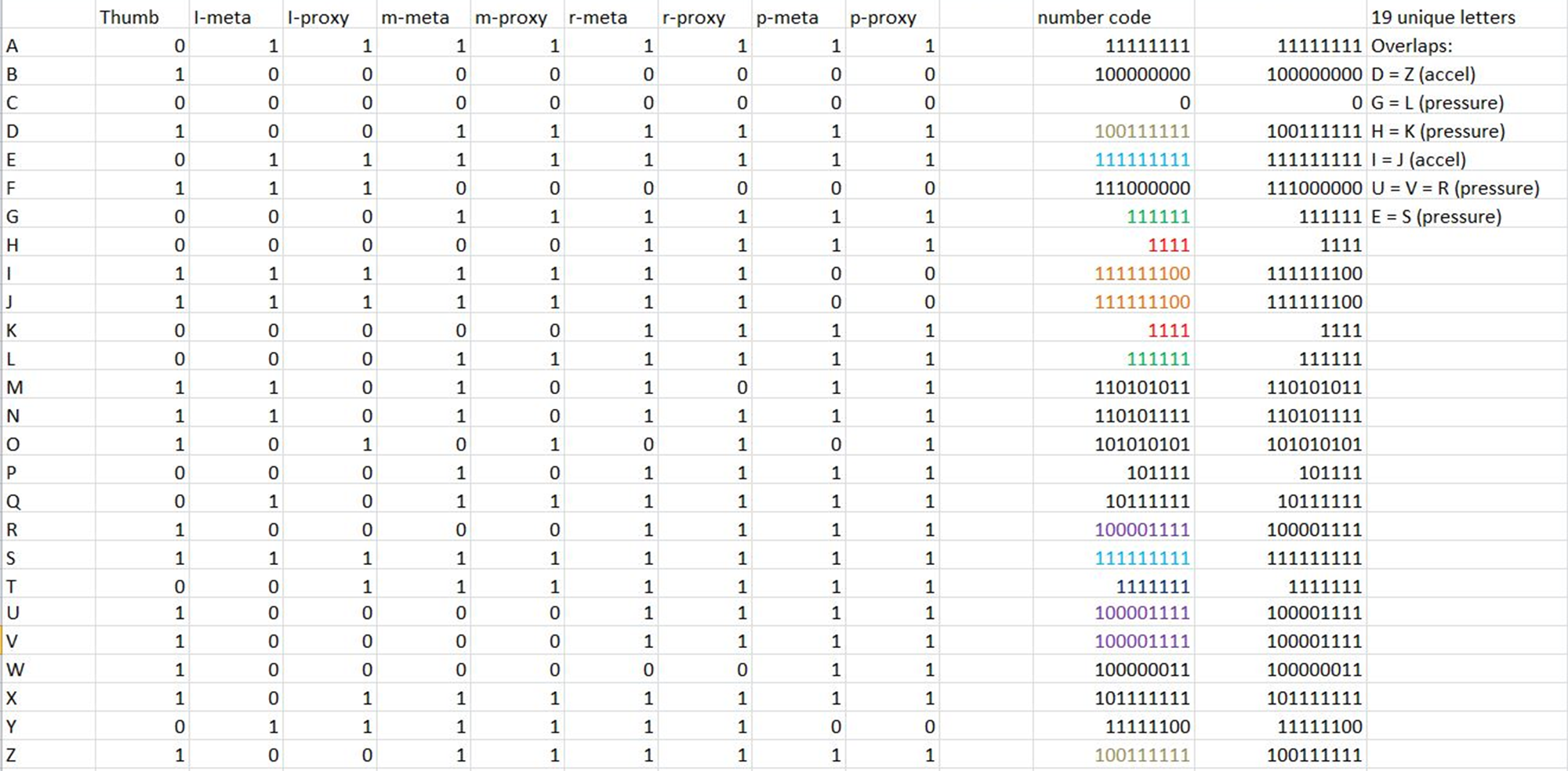

Supplement: S14 Fig — Key generation table indicating which letters correspond to which keys. The table also shows which letters have redundant keys and which type of hardware was used to differentiate those redundant letters. Letters were selected by monitoring the state of each sensor, assigning a 0 or 1 depending on the amount the finger was bent (0 for relaxed, 1 for bent). The individual numbers for each knuckle were then concatenated into a nine-digit code by summing powers of 10 (see code). For example, if the hand were completely relaxed, the code would read “000000000” and if a fist were formed, bending all knuckles, the code would be “111111111.” Each letter was assigned a nine-digit key. The table used to determine which letter would be assigned which key is shown below in S5 Fig. This image shows the table, the order of the sensors in building the key, and which letters have degenerate keys along with which hardware would be required to differentiate between those letters. (TIF) [file pone.0179766.s014.tif]

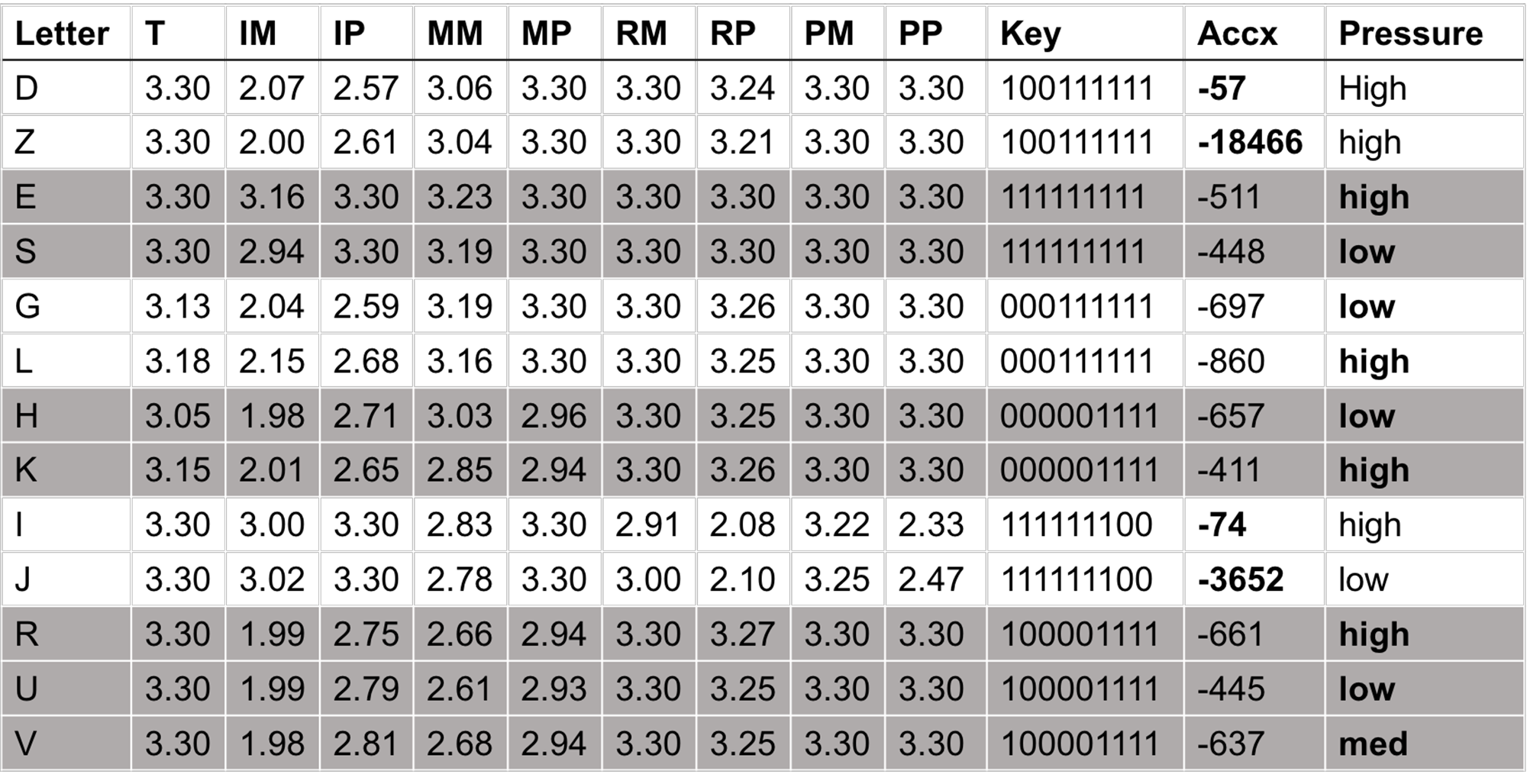

Supplement: S15 Fig — Table depicting the serial outputs of the parameters used to differentiate between letters with degenerate keys. A pressure sensor was used to differentiate between E/S, G/L, H/K, and R/U/V while an accelerometer was used to decouple the letters D/Z and I/J. The threshold for the x-acceleration was set to |3000|, while the pressure sensor was set to Pressure = 3.3 for high, 3.3 > Pressure ≥ 1.0 for med, and 1.0 > Pressure for low. (TIF) [file pone.0179766.s015.tif]

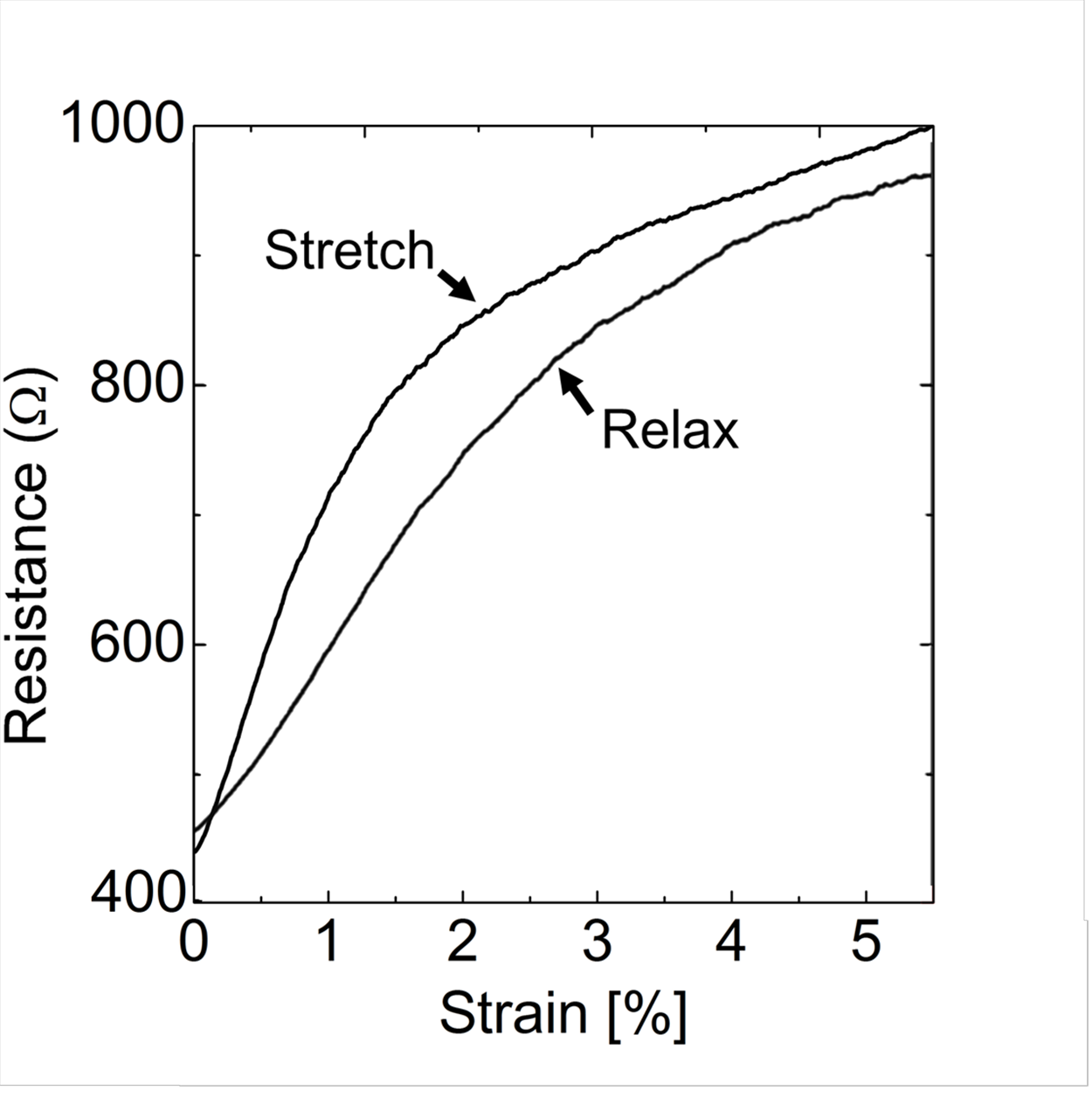

Supplement: S16 Fig — Hysteresis of the resistance vs. strain as the sensor was stretched from rest to 5.5%, the controllably released back to its initial length. (The drop in resistance at 5.5% is due to the pause of the machine and viscoelastic effects in the sensor). (TIF) [file pone.0179766.s016.tif]
